# Supplementary material for: Identification of Freezing-Responsive microRNAs and Their Targets in Chinese Jujube by Small RNA and Degradome Sequencing
Source: Int J Mol Sci. 2026 Sep 4;27(17):7912. doi: 10.3390/ijms27177912 (PMC13566890; doi:10.3390/ijms27177912)
Supplement: Supplementary file 1 [file ijms-27-07912-s001.zip › Supplementary Material S2/novel_miR_146.pdf]

Provisional ID : novel_miR_146
Score total : 268.5
Score for star read(s) : 3.9
Score for read counts : 261.7
Score for mfe : 1.3
Score for randfold : 1.6
Score for cons. seed :
Total read count : 525
Mature read count : 520
Loop read count : 0
Star read count : 5

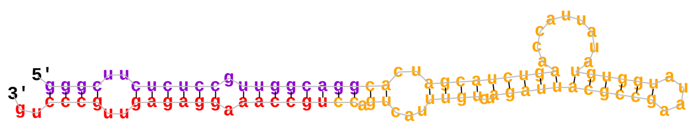

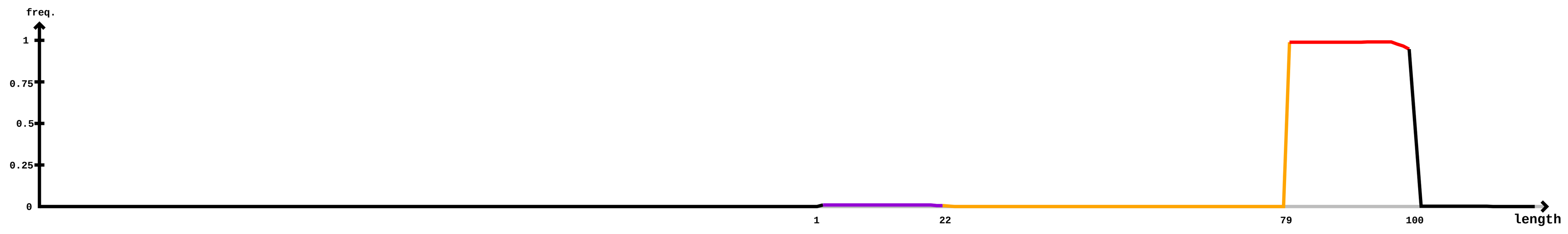

Star Mature

```
5'- ccauguauuccgcucaaaaucuagcacuuauaaaucuugugcucaaccgugaacaauagccaguaucaguuugaggcauagcaagagaaggagauagauaguacuucaauaauaagcaaaucagcuauagggcuucucuccguuggcaggcacuagcaucugaaccauuauaugugguauaagccgcauuagaucuguuuacugaccugccaaaggagaguugcccugugacugcuuuagcuuaauauag -3' obs
    ccauguauuccgcucaaaaucuagcacuuauaaaucuugugcucaaccgugaacaauagccaguaucaguuugaggcauagcaagagaaggagauagauaguacuucaauaauaagcaaaucagcuauagggcuucucuccguuggcaggcacuagcaucugaaccauuauaugugguauaagccgcauuagaucuguuuacugaccugccaaaggagaguugcccugugacugcuuuagcuuaauauag exp
    .....((((...(((....(((.((...........(((((((((.(.(((((........)).)))).)))).))))))).)))....)))...))))........(((.((((((((.(((.((((((((..((((((.((((((((((..(((((((((.........(((((....)))))))))))..))))...)).)))))))).))))))..)))))))).))).))).))))).)))..
```

read	reads	mm	sample
Aggcuucucuccguuggca	1	1	LT-3
gggcuucucuccguuggca	1	0	LT-1
gggcuucucuccguuggcagg	3	0	LT-3
ugccaaaggagaguugcU	1	1	LT-3
ugccaaaggagaAuugcc	1	1	LT-3
ugccaaaggagaguugcc	3	0	LT-2
ugccaaaggagaguugcc	2	0	LT-3
ugccaaaggagaguugccc	1	0	LT-3
ugccaaaggagaguugccc	5	0	LCK-3
ugccaaaggagaguugcccA	1	1	LT-2
ugccaaaggagaguugcccu	8	0	LCK-3
ugccaaaggagaguugcccu	1	0	LT-2
ugccaaaggagaguugcccug	146	0	LT-2
ugccaaaggagaguugcccug	7	0	JCK-3
ugccaaaggagaguugcccug	22	0	LCK-1
ugccaaaggagaguugcccug	44	0	LCK-3
ugccaaaggagaguugcccug	184	0	LT-3
ugNcaaaggagaguugcccug	3	1	LT-2
ugccaaaggagaguugcccug	12	0	JCK-2
ugccaCaggagaguugcccug	1	1	LT-2
ugccaaaggagaguugcccug	3	0	JCK-1
ugccaaaggagaguugcccug	48	0	LT-1
uAccaaaggagaguugcccug	1	1	LT-2
ugccaaaggagaguugcccug	25	0	LCK-2
ugccaaaggagaguugcccugA	1	1	LT-3
uugcccugugacugcuuuagc	1	0	LT-3
